# Conservation of oncofetal antigens on human embryonic stem cells enables discovery of monoclonal antibodies against cancer

**DOI:** 10.1038/s41598-018-30070-z

**Published:** 2018-08-02

**Authors:** Heng Liang Tan, Charlene Yong, Bao Zhu Tan, Wey Jia Fong, Jayanthi Padmanabhan, Angela Chin, Vanessa Ding, Ally Lau, Lu Zheng, Xuezhi Bi, Yuansheng Yang, Andre Choo

**Affiliations:** Bioprocessing Technology Institute, Agency for Science, Technology and Research (A*STAR), Biopolis, Singapore, Singapore

## Abstract

Monoclonal antibodies (mAbs) are used as targeted therapies against cancers. These mAbs kill cancer cells via various mechanisms of actions. In this study, human embryonic stem cells (hESCs) was used as the immunogen to generate a panel of antibodies. From this panel of mAbs, A19 was found to bind both hESC and various cancer cell lines. The antigen target of A19 was identified as Erbb-2 and glycan analysis showed that A19 binds to a N-glycan epitope on the antigen. A19 was elucidated to internalize into cancer cells following binding to Erbb-2 and hence developed as an antibody-drug conjugate (ADC). Using ADC as the mechanism of action, A19 was able to kill cancer cells *in vitro* and delayed the onset of tumour formation in mice xenograft model. When compared to Herceptin, A19 binds to different isoforms of Erbb-2 and does not compete with Herceptin for the same epitope. Hence, A19 has the potential to be developed as an alternative targeted therapeutic agent for cancers expressing Erbb-2.

## Introduction

Cancer which is characterized by abnormal cell growth is a major cause of death, killing over 8 million people globally^1^. The number of diagnosed cases is expected to double in the next two decades^2–4^. Conventional interventions to cancers include surgery, chemotherapy and radiotherapy^5–7^. Over the decades, cancer survival has increased due to advances in cancer treatments^1,8–10^. One such advancement is the development of targeted therapeutics with the use of monoclonal antibodies (mAbs).

The concept of antibodies serving as ‘magic bullets’ for cancer therapy dates back to their discovery in the late 19^th^ century^11,12^. With the discovery of tumour specific antigens in the mid-20^th^ century and the development of the hybridoma technology by Kohler and Milstein in 1975, mAbs rapidly emerged as a new class of targeted cancer therapeutics^1,3,11–13^. In addition to their specificity to the targets, antibodies have favorable pharmacokinetics and can be produced in standardized manufacturing processes^1,14–17^.

When antibodies bind to the targeted cells, they exert various effects on the tumour cells. The Fc-region of antibodies plays a critical role in immune cell activation and killing of tumour cells via antibody-dependent cell mediated-cytotoxicity (ADCC); and also in mediating tumour cell killing through complement-mediated cytotoxicity (CDC)^3,11,12,18,19^. Antibodies can cause vascular and stromal cell ablation, thereby affecting tumour cell growth. Alternatively, antibodies may neutralize or block the binding of growth factors to their respective receptors and subsequently inhibit cell proliferation^3,11,12,18^. They can also mediate direct cell killing by activating apoptotic pathways or via oncosis^1,11,12,19–23^. Antibodies are also used to deliver payloads such as drugs, radiation or cytotoxic agents to kill the tumour cells directly^3,11,12,19^.

Besides targeting cancer cells with antibodies, embryonic materials have also been investigated and utilized as alternatives to treat cancers. In separate studies, mice immunized with human fetal tissues or pluripotent stem cells (PSCs) exhibited strong protection against cancer tumour establishment and proliferation^24–26^. Cancer cells and embryonic materials share common cell surface markers and antigens known as oncofetal antigens. Some of the common oncofetal antigens used as biomarkers in oncology include cancer antigen 125 (CA125), CA19-9, prostate-specific antigen (PSA) and α-fetoprotein (AFP)^27–29^.

Tapping on the similarities in oncofetal antigen expression, our lab has successfully raised antibodies using human embryonic stem cells (hESCs) as immunogen^23,30–34^. One of the mAbs in the list, mAb 84, binds to the antigen Podocalyxin-Like Protein 1 (PODXL) on hESCs and kills the cells via oncosis^22,32^. PODXL is reported to be expressed in several cancers including breast, esophageal, lung and gastric adenocarcinoma, colorectal cancers, urothelial bladder and pancreatic cancers^35–43^. Another interesting candidate, mAb 8, is found to target the oncofetal antigen epithelial cell adhesion molecule (EpCAM), which is highly expressed in epithelial carcinomas and also expressed in many cancer types like breast, ovarian, colorectal adenocarcinomas and gastric cancers^33,44–50^. Another mAb, mAb-A4, which recognizes the glycan epitopes H type 1 and type 1 N-acetyllactosamine on hESCs, also binds to human ovarian and breast cancer cell lines but not to human normal cells^34^.

In this study, we report of another IgG1 from our hESC-immunization panel, mAb A19. A19 not only binds to undifferentiated hESCs by flow cytometry, it was found to also react with ovarian and breast cancer cell lines but exhibits low or no binding to normal cells. Via immunoprecipitation and mass spectrometry, the antigen target of A19 was identified as Erbb-2. Further investigation showed that A19 binds to N-glycan epitope on Erbb-2. In addition, A19 internalizes into cancer cells that have high expression levels of Erbb-2 and thus is useful as an antibody drug conjugate (ADC) to kill these cells *in vitro*. In preliminary *in vivo* model, the ADC is able to delay the onset of tumor formation. Our investigation suggests A19 to be a potential mAb to be used in immunotherapy.

## Results

### Binding of A19 to various cancer cell lines

A19 was raised against hESC in mice and the isotype was determined to be IgG1 (data not shown). Apart from staining strongly to hESC as determined by flow cytometry, A19 was also found to bind strongly to a range of breast and ovarian cancer cells lines and negligible to weak binding to normal cells (Table 1).

**Table 1:** Binding of A19 to various cancer cell lines.

Cell type	Cell line	Binding
hESC	HES-3	+++
Ovarian cancer	OVCAR3	+++
	OVCAR8	+++
	SKOV3	+++
	CaOV3	+
	HEY	+
	IGROV1	−/+
	OVCA432	−/+
	A2780	−/+
	HEYA8	−/+
	OV90	−
	OVCA433	−
	HEYC2	−
	OVCAR10	−
	TOV112D	−
Breast cancer	T47D	+++
	CAMA1	+++
	MCF7	++
	MDA-MB-231	++
	HS578T	+
Normals	Ovarian, IOSE523	−
	Breast, MCF10A	−
	Renal	−
	Human Esophageal Epithelial Cells	−/+
	Keratinocytes	−
	Hepatocytes	−
	Adult Mesenchymal Stem Cells (aMSC)	−
	Umbilical vein endothelial	−
	Red blood cells	−
	PBMCs	−
	Corneal stromal fibroblast	−
	Monocytes	−
	Lung fibroblast	−
	Foreskin fibroblast	−
	Embryonal Kidney	−
	Fetal MSC	−/+
	Umbilical cord	−/+
	hESC-derived MSC	+
	Pancreas duct	−

The cells were incubated with A19 on ice for 45 mins, washed and stained with secondary FITC. The binding of A19 is analysed by flow cytometry and binding is based on the population shift from the negative control as follows: − negative binding represents <20% cell population binding; −/+ low binding represents 20–40% cell population binding; + low-medium binding represents 40–60% cell population binding; ++ high-medium binding represents 60–80% cell population binding; +++ high binding represents >80% cell population binding. The grading of the cell population binding is represented as histograms in Supplementary Figure 1.

### Identification and Validation of A19’s target antigen

**Figure 1:**
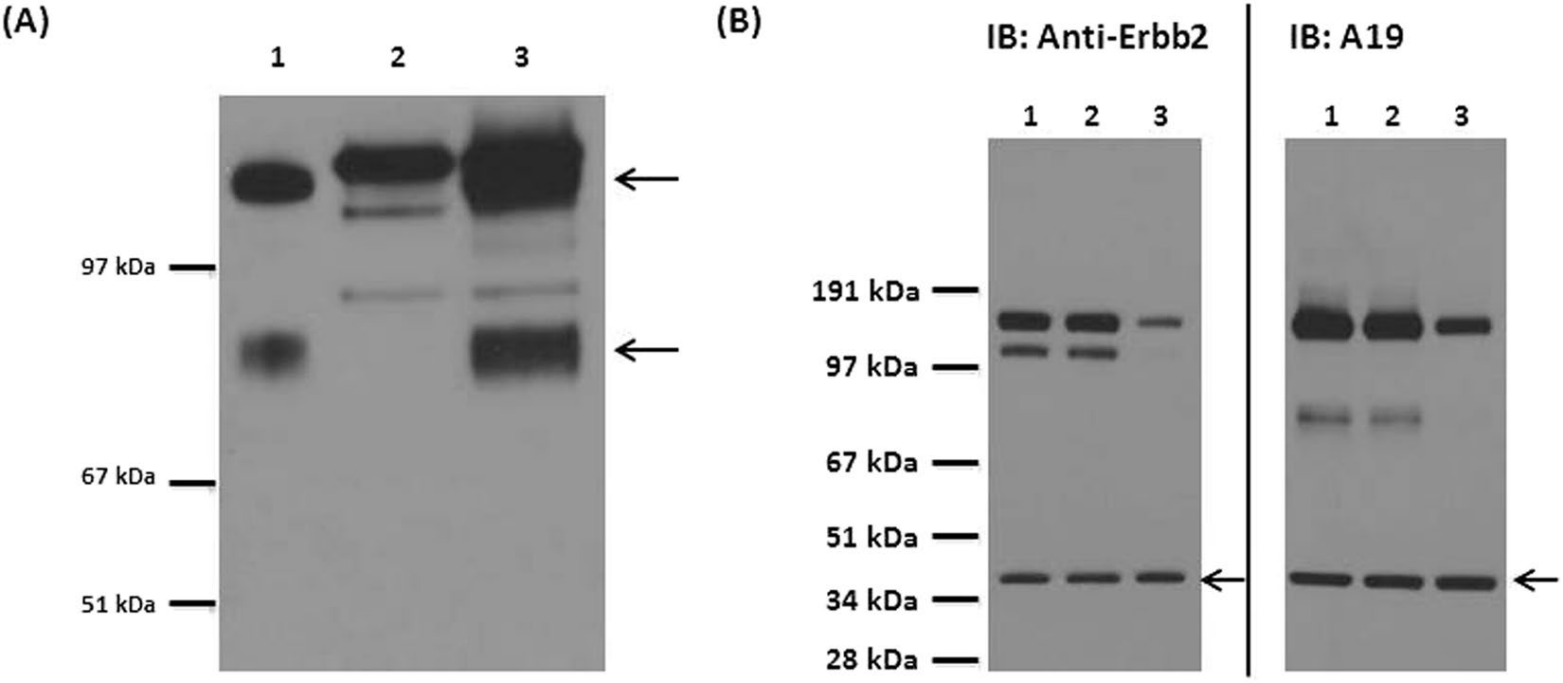
Identification and validation of antigen target. (**A**) Western blot of protein samples immunoblotted (IB) with A19. Lane 1; membrane fraction of SKOV3. Lane 2; IP with mAb A19 only (negative control). Lane 3; IP product – the 2 antigen bands are indicated by the arrows and were excised from a parallel SDS Page gel and the antigen targets identified via MS. Figure had been cropped to display protein bands >51 kDa and molecular weight markers were digitally added. Figure was converted to grayscale using Microsoft PowerPoint. Full length blot is presented in Supplementary Figure 2. (**B**) Validation of the antigen target as Erbb2. Lane 1; lipofectamine control, Lane 2; scramble control, Lane 3; knockdown sample. The samples were normalised at 20 μg per well and actin was used as a loading control (black arrows). Knock down of Erbb-2 was carried out using siRNA. Via western blot, both anti-Erbb-2 and A19 showed diminished binding in Lane 3 compared to the controls lanes (Lanes 1 and 2), confirming that the antigen target is Erbb-2. Figures had been cropped to display protein bands >28 kDa and molecular weight markers were digitally added. Figures were converted to grayscale using Microsoft PowerPoint. Full length blots are presented in Supplementary Figure 3.

The isolated membrane fraction from SKOV3 was separated by SDS-PAGE and immobilized onto a PVDF membrane by Western blot. When probed with A19, 2 antigen bands were observed after development between 67–97 kDa and above 97 kDa (Fig. 1A, Lane 1). We proceeded to identify the antigen targets by carrying out immunoprecipitation (IP) of the antigen from the isolated membrane fraction of SKOV3 with A19 immobilised on Protein G. The enriched antigens were detected via Western blot and the corresponding bands were excised from a parallel SDS-PAGE gel and identified via mass spectrometry (MS) (Fig. 1A, Lane 3). The upper band was identified as Isoform 4 of Receptor tyrosine-protein kinase Erbb-2 (Accession #P04626-4) and the lower band was identified as Receptor tyrosine-protein kinase Erbb-2 (Accession #F5H1T4). The peptide coverage of the peptides from the mass spectrometry spanned 39% of the entire protein for Isoform 4 of Receptor tyrosine-protein kinase Erbb-2 and 14% for Receptor tyrosine-protein kinase Erbb-2 (data not shown). Interestingly, Receptor tyrosine-protein kinase Erbb-2 is a truncated protein of Isoform 4 of Receptor tyrosine-protein kinase Erbb-2. To validate the antigen targets, a transient knockdown was carried out using siRNA targeting Erbb2 (Fig. 1B). The knockdown of Erbb2 was confirmed with a commercially available anti-Erbb2 (Lane 3). Similarly, diminished bands were observed in the knockdown sample when immunoblotted (IB) with A19 (Lane 3). The data confirms that the antigen targets of A19 are isoforms of Erbb2.

### Binding of A19 to a glycan epitope

**Figure 2:**
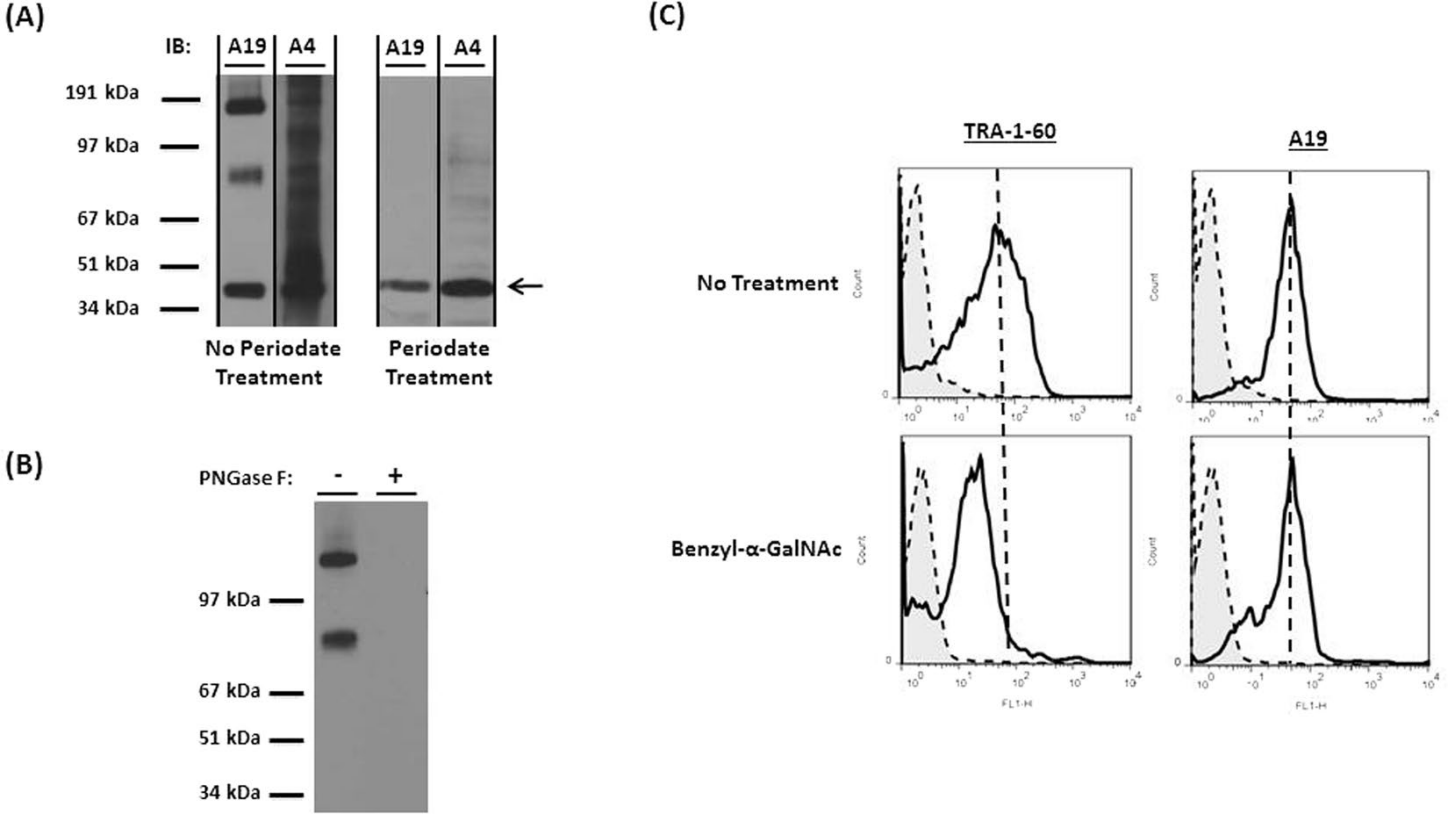
Analysis of A19’s glycan epitope. (**A**) SKOV3 membrane fraction was exposed to periodate oxidation and immuno-blotted with mAb A4 (positive control) and A19. Figures had been cropped to display protein bands >34 kDa and molecular weight markers were digitally added. Figures were converted to grayscale using Microsoft PowerPoint. Full length blots are presented in Supplementary Figure 4. (**B**) SKOV3 membrane fraction was digested with PNGase F to remove N-glycans and western blot was carried out. Figure had been cropped to display protein bands >34 kDa and molecular weight markers were digitally added. Figure was converted to grayscale using Microsoft PowerPoint. Full length blot is presented in Supplementary Figure 5. (**C**) hESC was cultured with 4mM benzyl-α-GalNac for 2 days. Through flow cytometry, a decrease in the binding of TRA-1-60 was observed for the benzyl-α-GalNac treated cells, confirming the inhibition of O-glycosylation. There was no decrease in A19’s binding to the cells for both conditions.

The antigen target of A19 was identified as Erbb-2. Erbb-2 is a glyco-protein with predicted N- and O- linked glycosylation sites (http://www.cbs.dtu.dk/services/NetOGlyc/, http://www.uniprot.org/uniprot/). To determine if A19 recognizes the carbohydrate or protein epitopes on Erbb-2, the immunoblot of SKOV3 membrane fraction was exposed to acidic periodate oxidation which cleaves the carbohydrate vicinal hydroxyl groups without altering the structure of proteins^51^. As a positive control, mAb A4, which was previously reported to bind to glycans^34^, showed no binding post periodate treatment (Fig. 2A). Similarly, binding of A19 to the expected 2 glycoprotein bands was not observed post periodate binding, suggesting that A19 is binding to glycan epitopes. The actin bands (indicated by the arrow) are controls to show that the structure of proteins are not altered by the periodate oxidation.

There are 2 main types of glycosylation. N-linked glycans attach to the asparagine or arginine side chains while O-linked glycans attach to proteins via the serine or threonine side chains^52^. To determine if A19 binds to N-glycans on Erbb-2, the membrane fraction of SKOV3 was digested with PNGase F to remove N-glycans on glyco-proteins and Western blot carried out^53–55^. In Fig. 2B, the binding of A19 was abolished post-PNGase treatment, indicating that A19 binds to N-glycans. To investigate if A19 binds to O-glycans on Erbb-2, benzyl-α-GalNac was used to inhibit O-glycosylation in hESC culture^23^. The inhibition of O-glycosylation is confirmed by the loss of binding of TRA-1-60 which binds to a specific type 1 lactosamine epitope^23,56^. However, there was no difference in A19 binding in both the control and benzyl-α-GalNac treated hESC lysate. Taken together, the data shows that A19 binds to N-glycans on Erbb-2.

### Mechanism of Action (MOA) of A19

**Figure 3:**
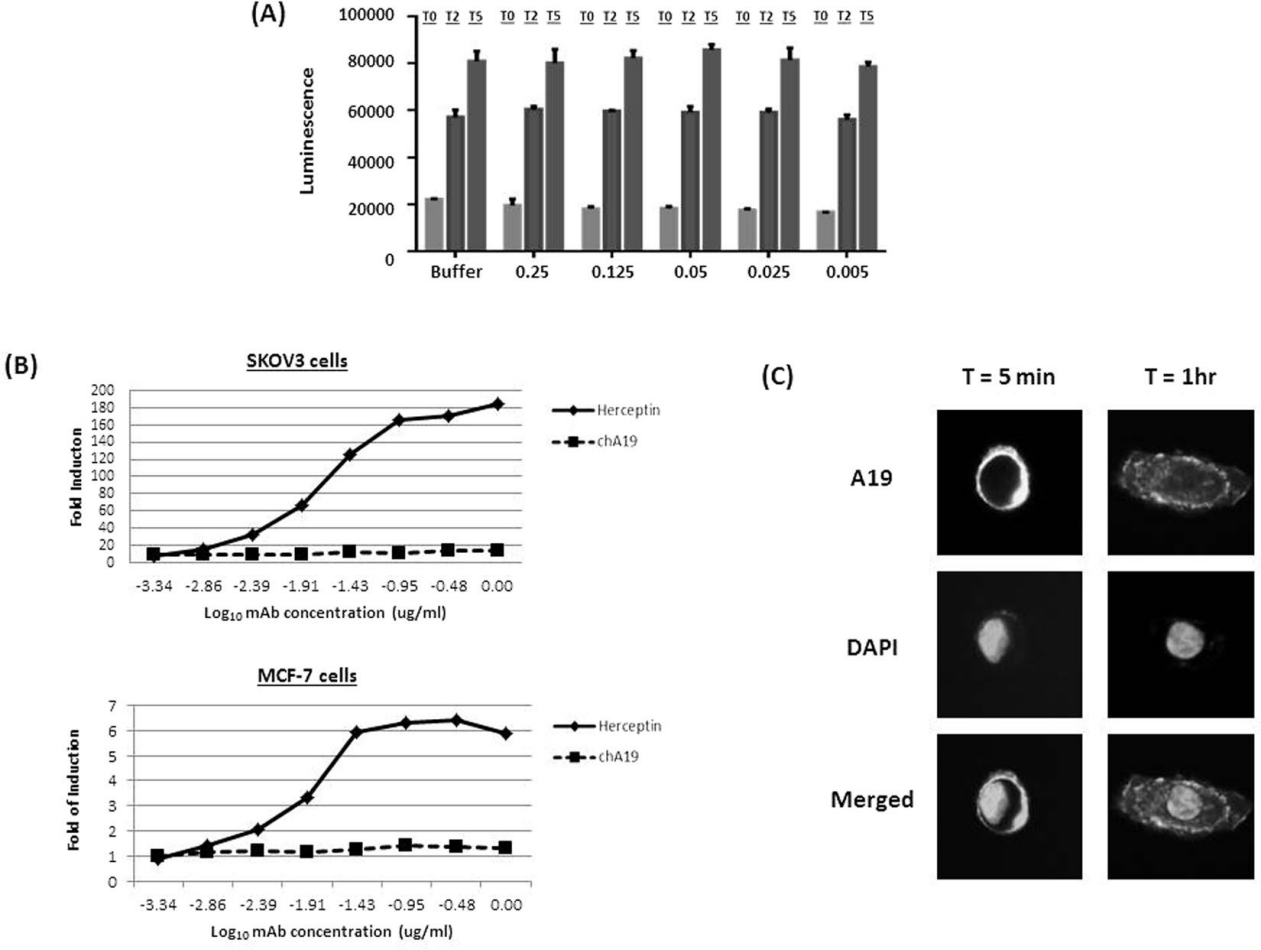
Determining the mechanism of action of A19. (**A**) A19 was spiked into SKOV3 at various doses and viable cell number measured through CTG at Day 2 (T2) and Day 5 (T5). The number of viable cells at various doses of A19 was comparable to the control at both T2 and T5. Values are average of 6 wells ± standard deviation. (**B**) *In vitro* ADCC activity was measured as fold induction of the NFAT pathway using an ADCC reporter bio-assay (Promega). Chimerised A19 (chA19) did not exhibit any ADCC activity when cultured with either ovarian and breast cancer cells. The isotype control, Herceptin, is used as a positive control. (**C**) Via immunofluorescence staining, A19 internalizes into SKOV3 cells.

We proceeded to determine if A19 has any effect on the growth of cancer cells. We first investigated if A19 could inhibit the growth of the cells as a naked antibody. SKOV3 cells were spiked with various doses of A19 and viable cell number was measured at Day 2 (T2) and Day 5 (T5) (Fig. 3A) using the CellTiter-Glo® Luminescent Cell Viability Assay kit. A19 did not display any inhibitory effect on the cells even at a high dosage of 0.25 mg/ml. Next, we chimerised A19 (chA19) with a human IgG1 backbone and tested if chA19 exhibited antibody-dependent cell-mediated cytotoxicity, ADCC, on SKOV3 and MCF-7^57^. The ADCC activity was evaluated via a gene reporter assay which uses engineered Jurkat cells expressing the human Fc-gamma IIIA receptor coupled to an ADCC NFAT (nuclear factor of activated T-cells) signalling pathway as readout. Herceptin which is also an IgG1 and known to elicit ADCC was used as a positive isotype control^58,59^. However, chA19 did not exhibit any ADCC.

We then tested if A19 could be internalized into the cells after binding. When A19 was incubated with SKOV3 cells for 5 min, surface binding was evident as the cells were stained brightly as a ring structure around the cells (Fig. 3C). After an hour of incubation, punctate fluorescence staining was observed across the cell confirming that the mAb is internalized into the cells. Internalization of chA19 into MCF7 cells was also captured in real-time (Supplementary Video 1). The chA19 was biotinylated and conjugated to streptavidin-pHRodo, a dye which fluoresces maximally when it enters the endosome where the pH is low. The video captured the internalization process of the mAb, which occurred over a 1 hour period.

### A19 as an Antibody-Drug Conjugate (ADC)

**Figure 4:**
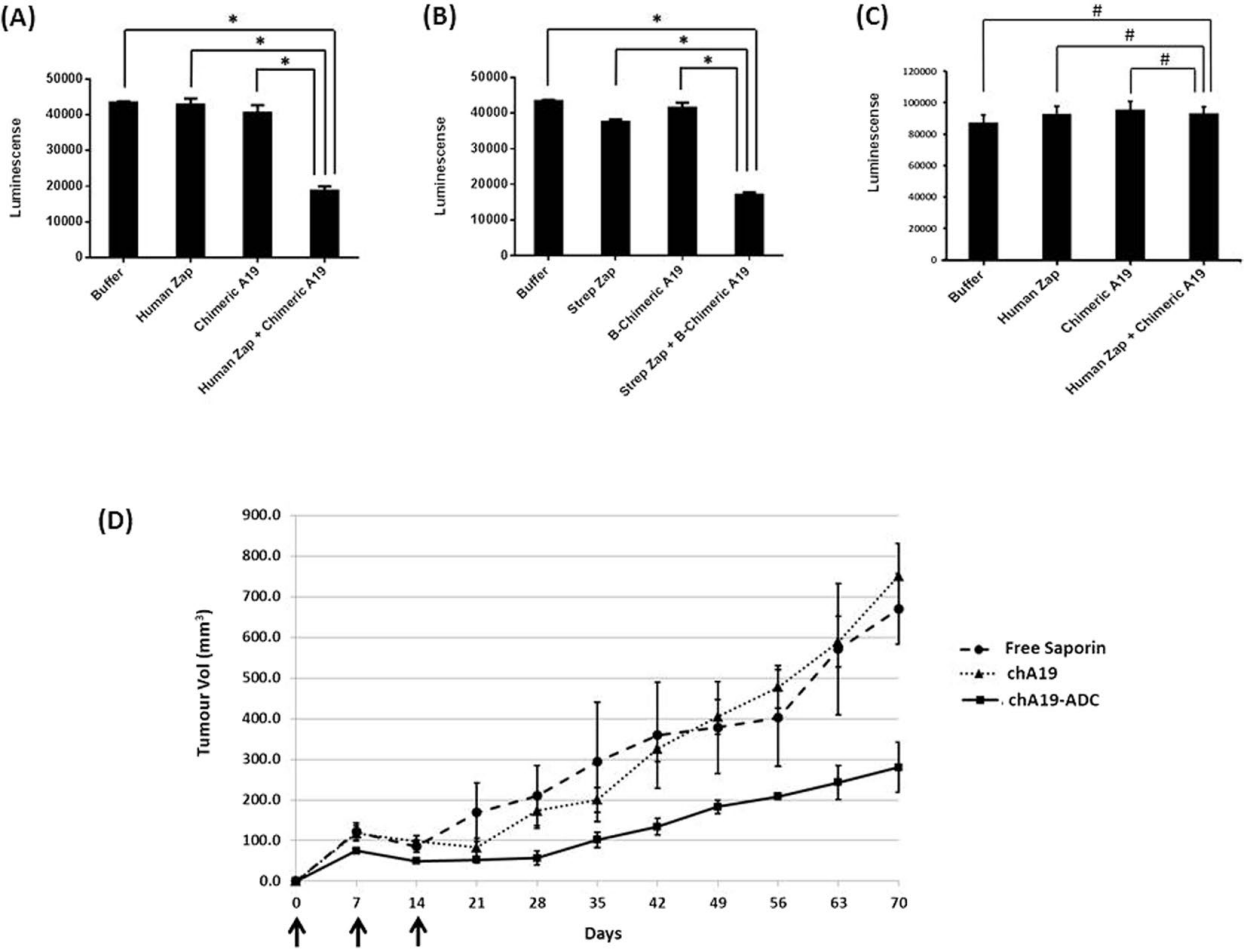
A19 as an Antibody-Drug Conjugate. (**A**) chA19 is conjugated to the toxin saporin via a secondary mAb or (**B**) via streptavidin-biotin affinity. As an ADC, A19 kills the cancer cells *in vitro*. *p < 0.01. (**C**) As an ADC, A19 does not kill OVCAR10. ^#^*p* > 0.01 (**D**) Nude mice were implanted s.c. with 5 × 10^6^ SKOV3 in Matrigel. Three doses of A19-ADC (37.5 mg/kg per dose) was administered 1 week apart, intraperitoneally as indicated by the arrows. n = 4 animals per group; bars represents standard error. The A19-ADC suppresses tumour growth *in vivo*.

Since A19 is able to bind specifically to Erbb2 and internalize into cells, it could potentially be developed into an ADC. To test this, we pre-incubated anti-human secondary-saporin with chA19 at a 1:1 molar ratio. The ADC complex was subsequently spiked into SKOV3 cells that were seeded overnight, at a primary antibody concentration of 2 μg/ml^60^. Viable cell number was determined by quantitation of ATP present after 72 h. From Fig. 4A, the number of viable cells that were treated with the secondary-saporin (Human Zap) and chA19 alone were comparable to the buffer control. However, the cells treated with ADC complex showed a decrease in cell number. Complementary assay was also carried out to validate the efficacy of the ADC complex. The cells in the various conditions were scaled-up accordingly to 24-wells each and the number of viable cells was determined. From Supplementary Figure 6, the viabilities of the cells for all conditions were high. However, the number of total cells post-treatment with the ADC complex was lower than the other 3 controls, consistent with the CTG results. Next, we proceeded to biotinylate the antibody and complexed it directly to streptavidin-saporin (Strep Zap). Similarly, the ADC complex showed a significant decrease in viable cell number although Strep-Zap exhibited a low percentage of cytotoxicity (Fig. 4B). To determine if there are off-target effects, human Zap and chA19 were incubated with a non-binding cell line OVCAR10. No apparent cytotoxicity was observed (Fig. 4C). Putting these data together, chA19 demonstrated *in vitro* efficacy and can be used as an ADC.

We next tested the ADC effect in an *in vivo* model whereby 5 × 10^6^ SKOV3 cells were implanted subcutaneously (s.c.) in NUDE mice and the ADC, chA19-Biotinylated–Streptavidin-Saporin, administered intraperitoneally (Fig. 4D). In this pre-emptive model, the ADC was given at t=0, week 1 and week 2, at a dose of 37.5 mg/kg (22). The controls were free saporin and naked chA19. By the end of 10 weeks, mice administered with the ADC saw a 60% reduction in tumour size compared to the control groups. Hence A19-ADC was able to suppress tumour growth.

### A19 does not compete with Herceptin

**Figure 5:**
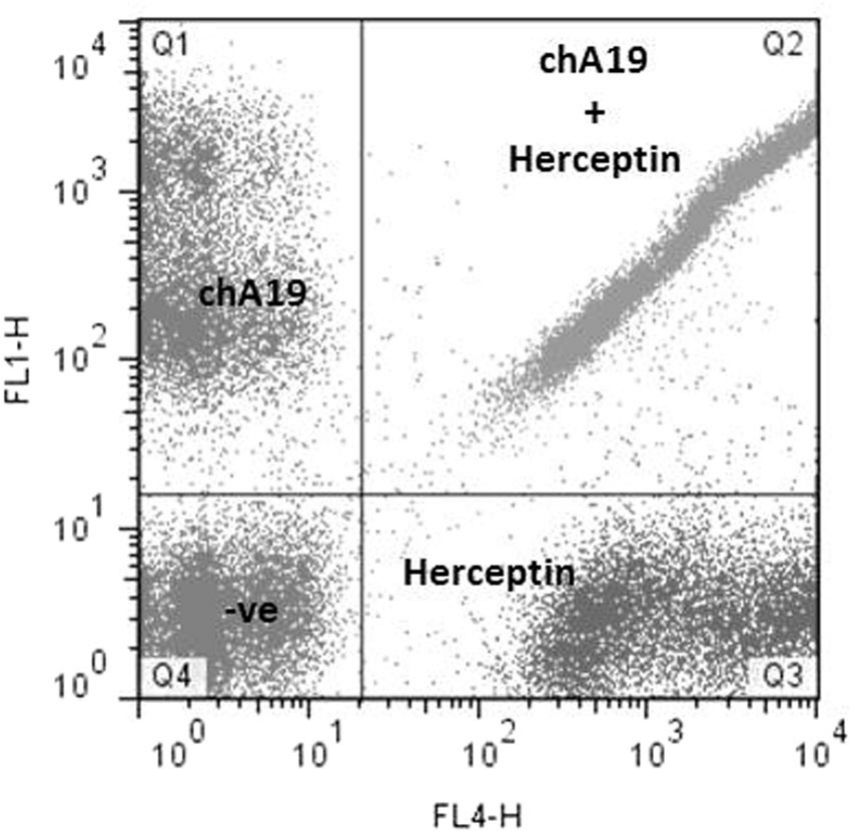
Competitive binding between A19 and Herceptin. A19 and Herceptin were individually conjugated to Alexafluor 488 and APC respectively. Single stains showed that the antibodies bind to the cells (Q1 for A19, Q3 for Herceptin). When the antibodies were incubated together, they do not compete with each other (Q2).

As A19 binds to Erbb-2, we investigated if it competes for the same epitope with Herceptin. Both chA19 and Herceptin were conjugated directly to Alexa Fluor 488 and APC respectively. SKOV3 cells were either single or double stained with the mAbs, and the readout analysed through flow cytometry (Fig. 5). Individually, the mAbs bind strongly to the cells (chA19-Q1 and Herceptin-Q3). When incubated together, binding of both mAbs to the same cell population was not affected (Q2). This shows that A19 and Herceptin bind to different epitopes on Erbb-2.

## Discussion

In this study, a panel of mAbs was raised using hESC as an immunogen. From there, mAb A19 was found not only to bind strongly to hESC, it also bound strongly to various ovarian and breast cancer cells. This is not surprising since embryonic and cancer cells share common biomarkers known as oncofetal antigens. Via IP/MS, the antigen target of A19 was found to be Erbb-2 and the mAb detects 2 protein bands on Western blot: Isoform 4 of Receptor tyrosine-protein kinase Erbb-2 (higher molecular weight) and Receptor tyrosine-protein kinase Erbb-2 (lower molecular weight). Isoform 4 of Receptor tyrosine-protein kinase Erbb-2 belongs to the family of Receptor Tyrosine Kinase with 6 isoforms produced by alternative splicing and alternative initiation. Interestingly, the lower molecular weight protein detected by A19, Receptor tyrosine-protein kinase Erbb-2 is a truncated protein of Isoform 4 of Receptor tyrosine-protein kinase Erbb-2; missing a.a 1–15 and a.a 619–1240. Erbb-2 belongs to the family of human epidermal growth factor receptor (HER) and plays a central role in many processes (especially in cancers) such as cell proliferation, survival and metastasis^61,62^. The expression of Erbb-2 on hESC was also previously reported by *Wang et al*. and it was shown that inhibition of the Erbb-2 signalling severely inhibited hESC proliferation and promoted apoptosis^63^.

Herceptin binds to Erbb-2 and is used widely as therapeutics in the clinics to treat HER2 positive breast cancers and gastric cancers^2,64^. We proceeded to compare A19 with Herceptin. Interestingly, A19 and Herceptin bind to different isoforms of Erbb-2 (Supplementary Figure 7). A19 binds to 2 isoforms while Herceptin binds to 3 isoforms. When we screened both mAbs against normal cell lines, we observed Herceptin to be non-specific than A19 (Supplementary Table 1).

Several mechanisms of action have been proposed for Herceptin. Reports suggest that the main MOA are inhibition of Erbb-2 signaling and ADCC^11,12^. Herceptin was found to internalize into cells and was developed into an antibody drug conjugate, Kadcyla^65^. Kadcyla or ado-trastuzumab amtansine is a conjugate of Herceptin and a cytotoxic moiety, DM1 which is a derivative of maytansine. Kadcyla carries an average of 3.5 DM1 per molecule of antibody^66^. Kadcyla showed good *in vitro* and *in vivo* efficacy and the median progress-free survival (PFS) was 14.2 months compared to the PFS of 6.2 months with Herceptin plus docetaxel^67^. Despite favourable efficacy, most patients treated with Kadcyla eventually progresses. Also, some HER2-positive breast cancers are primarily non-responsive or are minimally responsive to Kadcyla. One of the plausible reasons for this resistance is the poor internalization of the HER2-Kadcyla complexes^66^. Here, we have shown that A19 binds to N-glycans on Erbb-2 and to a different epitope compared to Herceptin. We have also demonstrated that A19’s MOA is internalization. As an ADC, although the chimeric A19 was sub-optimally conjugated in our lab, it was able to suppress the development of the xenografts. The next step would be to directly conjugate A19 with amtansine to enable a better comparison. Potentially, A19 could be developed as an alternative targeted therapeutic drug to HER2-positive cancers.

## Materials and Methods

### Cell culture

The hESC line HES-3 was cultured as described in Choo *et al*.^32^. The ovarian cell lines were a gift from Dr. Ruby Huang (Cancer Science Institute, Singapore). Other breast cell lines were obtained from the American Type Culture Collection (ATCC) and the National Cancer Institute 60 (NCI-60) panel of cancer cell lines. Cancer cells were cultured at 37 °C with standard culture media recommended by ATCC.

### Generation of mAbs

The generation of mAbs is as described in Choo *et al*. and in accordance to Biopolis Institutional Animal Care and Use Committee Approval 151005^32^. Briefly, 6-week old female BalbC mice were immunized with 1 × 10^6^ HES-3 cells/mice suspended in phosphate-buffered saline (PBS-) mixed with adjuvant (Sigma). Immunization was carried out weekly for 5 weeks. B-cells were harvested and fused with SP2/0 mouse myeloma cells using the ClonalCell-HY Hybridoma Cloning Kit (Stem Cell Technologies Inc). Hybridomas were isolated 10–14 days after plating and cultured in 96-well containing Medium E. Culture supernatant from each hybridoma clone was collected and reactivity to the various cell types was assessed by flow cytometry.

### Isotyping

Isotyping was performed with Mouse Monoclonal Antibody Isotyping kit from Roche (Roche, #11493027001). The protocol was carried out according to manufacturer’s instructions. Briefly, the pellet in the tube was reconstituted with 150 µl of hybridoma culture supernatant. The solution was thoroughly mixed by vortexing before adding the isostrip. The results were analyzed after 10 min of incubation in room temperature.

### Flow Cytometry Analysis

Cells were harvested using trypsin (Invitrogen, USA) to obtain a single cell suspension. Approximately 2 × 10^5^ cells in 10 μL of ice-cold 1% bovine serum albumin (BSA) (Sigma-Aldrich, USA) in phosphate buffered saline (PBS, Invitrogen, USA) were incubated in 100 μl of hybridoma culture supernatant containing monoclonal antibody A19 or 5 μg of purified mAbs for 45 minutes at 4 °C. Cells were then washed with ice-cold 1% BSA/PBS, and incubated with polyclonal goat anti-mouse immunoglobulin conjugated with fluorescein isothiocyanate (FITC) at a dilution of 1:500 (DAKO, Denmark) for 15 min in the dark. Cells were then washed with ice-cold 1% BSA/PBS and resuspended in 200 μl of 1% BSA/PBS for analysis on FACScalibur flow cytometer (BD Biosciences, USA).

### Membrane Protein Extraction

Adherent cells were scraped in PBS (Invitrogen, USA) and centrifuged at 3000 rpm for 5 min. The cells were washed in ice-cold PBS (Invitrogen, USA) and centrifuged at 3000 rpm for 5 min. The resulting pellet was resuspended in Homogenize Buffer Mix (BioVision, USA) and sonicated using Misonix Sonicator 3000 under the following conditions: a total process time of 5 min consisting of repeated cycles of 5 sec pulse on and 10 sec pulse off. The resulting homogenate was transferred to a 1.5 ml microcentrifuge tube and centrifuged at 700 g for 10 min at 4 °C to remove debris. The supernatant was then collected and centrifuged at 10,000 g for 30 min at 4 °C. The resulting supernatant was discarded and the pellet containing membrane protein extract was collected for subsequent analysis.

### Immunoprecipitation (IP)

Membrane protein was solubilized with 2% Triton in PBS. Immunoprecipitation was carried out using the Phynexus instrument (Phynexus Inc, California, USA), loaded with Protein G tips (Phynexus Inc, #PTR 92-05-02). The automated program allowed sequential incubation with either A19 or Herceptin, solubilized protein samples and washing buffers. Low pH elution was performed at the final step and the eluted sample was neutralized before use.

### SDS PAGE Gel and Western Blot

The samples were boiled at 95 °C after adding 5X sample loading dye and subjected to SDS-PAGE using 4–12% gradient NuPAGE Bis-Tris gel (#NP0335 Box) with 1X MOPS buffer (#NP001) (Life Technologies). The proteins were separated at 110 V for 1 h. The samples were prepared in duplicates, one set used for Western blot transfer onto PVDF membrane and the other for silver staining. The membrane blot was blocked with 5% low fat milk for 30 min before incubating overnight at 4 °C with diluted culture supernatant from the primary antibody (1:3) with blocking buffer. Blots were washed with 0.1% Tween in PBS, and incubated with horseradish peroxidase (HRP) conjugated anti-mouse or anti-human Ig (1:10,000, DAKO) at room temperature for 1 h. Finally, the blots were developed using chemiluminescence, ECL prime Western blotting detection reagent (GE Healthcare, #RPN2232). The protein band on the silver stained gel that corresponded to the Western blot was excised and digested with trypsin prior to antigen target identification using mass spectrometry (LC/MS-MS). For target validation, A19 and commercial antibodies against Erbb-2 (Herceptin/Abcam) were diluted 1:100 for IP and 1:1,000 for Western blotting.

### siRNA Knockdown

To validate the identity of target antigen, knockdown of target antigen was carried out with siRNA against Erbb-2 (Ambion, #103546) using Lipofectamine RNAiMAX according to the transfection protocol provided by the manufacturer. Scrambled siRNA was used as the negative control. Briefly, 1 × 10^5^ SKOV3 cells were seeded into 6-well plate and allowed to adhere overnight. Lipofectamine was added in equal volume (1:1) to separate tubes containing scrambled and Erbb-2 siRNA and allowed to stand for 20 min at room temperature. Culture media was aspirated from the 6-well plate and replaced with 3 ml/well of fresh media. 200 μl of the reagent mix was added dropwise into the respective wells and gently swirled to allow even distribution throughout the wells. The cells were incubated at 37 °C for 48 hr. The cells were harvested by scrapping and lysed with 2% Triton in PBS. Total protein concentration was quantified with DC Protein Assay (Bio-Rad Laboratories) and Western blot carried out as previously described.

### PerIodate

Proteins were resolved by SDS-PAGE and transferred onto PVDF membranes. The membranes were rinsed twice with washing buffer made up of 100 mM sodium acetate (Merck, Germany) at pH 4.5. Subsequently, the membranes were incubated with 100 mM sodium meta-perIodate (Sigma-Aldrich, USA) in washing buffer for 15 min in the dark. The process was repeated with fresh sodium meta-perIodate for another 15 min. Sodium acetate was added into the control instead of sodium meta-perIodate. After incubation, membranes were rinsed 4 times with washing buffer, followed by PBS. The membranes were then incubated with 0.5 M sodium borohydride (Sigma Aldrich, USA) for 30 min at room temperature. After incubation, the membranes were rinsed once with PBS and blocked in 5% milk in PBS-Tween for 30 min at room temperature. Thereafter, the blots were probed with primary and HRP-labelled secondary antibodies and detected via chemiluminescence.

### PNGase Digestion

PNGase digestion was carried out according to manufacturer’s protocol (New England Biolabs). Briefly, 10–20 µg of glycoprotein was first denatured in 1X glycoprotein Denaturing Buffer at 95 °C for 10 min. Denatured proteins were treated with 1 μl sialidase at 37 °C. Subsequently, 1X G7 Reaction Buffer and 10% NP-40 were added and the mix was digested with 2 μl of PNGase F at 37 °C for 1 h. Digested proteins were subsequently resolved on SDS-PAGE and transferred to Western blot. Controls were set up without the inclusion of enzyme.

### Inhibition of O-linked Glycosylation in hESC

Four days after passaging, hESC in culture were spiked with optimized amount of Benzyl-α-GalNac in conditioned media (CM) and incubated for 48 hr. For the negative control, hESC were fed with CM alone or CM with the same volume of DMSO as the inhibitor. Cells were trypsinized and resuspended as single cell suspension in 1% BSA/PBS. Flow cytometry analysis was carried out as described previously.

### CellTiter-Glo (CTG) Luminescent Assay

Cells were seeded (1000 cells/90 µl/well) to 96-well plates (black, clear flat bottom) in culture media and incubated overnight at 37 °C, 5% CO_2_. Working stocks of mAb or mAb conjugated with toxins were prepared in varying concentrations accordingly and added in volumes of 10 µl to the cultures. The cultures were incubated for another 72 h at 37 °C, 5% CO_2_. Metabolically active cells were measured based on the presence of ATP, using the CellTiter-Glo (CTG) Luminescent Cell Viability Assay kit (Promega). 100 µl of CTG substrate was added to each well and incubated for 15 min in the dark at RT on a shaker. Luminescence was measured using TECAN M2000. Experiments were carried out minimally twice (2 biological repeats) with 6 technical replicates for each condition.

### Cell Counting and Viability Assay

Cells were scaled up in 24-wells. Working stocks of mAb or mAb conjugated with toxins were prepared in varying concentrations accordingly and added to the cultures. The cultures were incubated for another 72 h at 37 °C, 5% CO_2_. To carry out the cell count and viability assay, the supernatants were collected, cells washed with PBS and trypsinised for 5 min. The cells harvested were added to the respective supernatant, centrifuge and supernatant discarded. The cell pellet was resuspened in 200 µl of PBS. To 38 µl of cell suspension, 2 µl of Solution 13 (Chemometec) was added and mixed via gentle vortex. The final cell suspensions were loaded into the chambers of NC-slide A8 and cell counts and viabilities were measured using the NucleoCounter NC-3000 (Chemometec).

### Chimerization of A19

Chimerization was done in-house by the Animal Cell Technology group at BTI. The construct was expressed in DG44-CHO cells and the cultures were maintained in BTI’s proprietary serum-free media^68^.

### Purification of mAbs

Purification of both mouse and chimeric mAbs was done using the ÄKTA Explorer 100 (GE Healthcare) system. Cultured supernatants were passed through Protein A chromatography (Tosoh; Toyopearl AF-rProtein A-650F) and ion exchange chromatography (Bio-Rad; UNOspher Q). The purified mAbs were evaluated on a Superdex200 PC 3.2/30 column (GE Healthcare) using a high performance liquid chromatography system (Shimadzu). Antibody concentration was determined by absorbance at *A*_280_ using Nanodrop 1000 (Thermo Fisher Scientific).

### Antibody-dependent cell-mediated cytotoxicity assay

ADCC activity was measured using a reporter bioassay (Promega; ADCC Reporter Bioassay, #G7010). The ADCC bioassay was carried out according to the manufacturer’s protocol. Briefly, cells were seeded at 5,000 cells per well in a 96-well clear bottom black tissue culture plates (Corning; #3904) in low 4% IgG-serum (Promega; #G711A) containing media. Serial dilutions of primary antibody were incubated in triplicate wells for approximately 15 min at 37 °C, 5% CO_2_. Following incubation, engineered effector cells were added to the wells at approximately 150,000 cells per well. After more than 5 h (or as indicated in results), Bio-Glo Luciferase Assay Substrate (Promega; #G719A and #G720A) was added to the wells and luminescence was measured using the Infinite 200 microplate reader (Tecan). Experiment was carried out twice (2 biological repeats) with 3 technical replicates for each condition.

### Internalization Studies

Biotinylated mAbs were incubated with equimolar of pHRodo Red Avidin (Thermo Fisher Scientific, #P35362) in the dark and on ice for 5 min prior to use. Conjugated mAbs (5 ug) was added to the cells and incubated in the dark and at room temperature for 2 h before analysis on the FACS Calibur via the FL2-H channel. Real time visualization of the internalization was carried out by video capture on the DeltaVision (GE Healthcare Life Sciences).

### Immuno-Fluorescence

Cells were trypsinized, seeded at 2,000 cells/well on two 24-well plates (Plate 1 and Plate 2) and left overnight in the incubator at 37 °C, 5% CO_2_. Both plates of cells were pre-chilled by washing twice with fresh cold media and topped up with 1 ml of cold media. Primary antibody was then added into the wells (final concentration of 4 µg/ml). For the 1st plate, incubation was carried out on ice for 5 min. For the 2nd plate, incubation was carried out at 37 °C for an hour to facilitate internalization. After the primary mAb incubation, both plates were washed twice with cold PBS and subsequently fixed with 4% Paraformaldehyde/PBS for 15 min. The cells were washed twice with cold PBS and permeabilized with 0.5% Triton-X/PBS for 10 min. The washing was repeated and cells were blocked with 10% Fetal Bovine Serum/PBS for 10 min. The cells were washed twice with PBS and incubated with anti-mouse Alexafluor 488 and DAPI (Thermo Fisher Scientific) for 30 min in the dark. Excess dyes were washed off with PBS and 500 µl 1% BSA/PBS was added to each well before imaging.

### Antibody Drug Conjugates (ADCs)

Primary mAbs were complexed with appropriate secondary antibody conjugates: mAb-ZAP or HUM-ZAP (Advanced Targeting Systems), at 1:1 molar ratio for 15 min at room temperature before spiking into the cultures. Alternatively, mAbs were biotinylated using the EZ-Link Sulfo-NHS-Biotin kit (Thermo Fisher Scientific) prior to incubating with Streptavidin conjugated Saporin (1:1 molar ratio) for 20 min at room temperature.

### Biotinylation of mAbs

The mAbs were biotinylated using the EZ-Link Sulfo-NHS-Biotin kit (Thermo Fisher Scientific). Briefly, 50 µl of Biotin Reagent was added to 1 ml of mAb (2 mg/ml in PBS) and incubated at room temperature for 30 min. Non-reacted biotin was removed by dialysis.

### *In Vivo* Model

The antibody drug conjugate complex was prepared by adding biotinylated A19 to Streptavidin conjugated Saporin (Advanced Targeting Systems) as described earlier. For the animal model, the pre-emptive model was adopted. Each nude mouse was injected in the right flank, subcutaneously, with 5 × 10^6^ SKOV3 cells in 100 µl volume PBS/matrigel (1:1 volume; BD Matrigel Matrix, #354234). The drug (37.5 µg per dose) was administered intra-peritoneal at Day 0, 7 and 14. Tumour size was monitored over 70 days. Each group consisted of 4 mice.

### Conjugation of mAbs to Fluorophores

Antibodies A19 and Herceptin were conjugated to Alexafluor 488 and Allophycocyanin (APC) respectively using the LYNX Rapid Conjugation Kit (AbD Serotec) according to manufacturer’s protocol. Briefly, 100–150 µg of antibody was used for every 100 µg of fluorophore. To the antibody sample, 1 µl of the Modifier reagent was added to every 10 µl of antibody and gently mixed. The mixed antibody-modifier solution was added into the supplied vial and the lyophilized LYNX reagent was resuspended by gentle pipetting of the solution twice. The antibody mix was incubated at room temperature for 3 h. After the incubation, 1 µl of Quencher was added to every 10 µl antibody used. The final solution was left to stand for 30 min before use.

### Competitive Assay

Single cell suspension of SKOV3 (0.5 × 10^6^ cells per 100 µl 1% BSA/PBS) were incubated individually or dually with 5 µg of conjugated mAbs on ice for 30 min. The cells were washed and resuspended in 200 µl buffer. A19 and Herceptin binding to cell populations were analysed on the FACS Calibur via the FL1-H and FL4-H channels respectively.

### Statistical analysis

All tests were performed using EXCEL to determine significant differences including mean and s.e.m. with independent sample two-tailed *t*-tests. *P*-values < 0.01 were considered significant.

## Electronic supplementary material

Supplementary Video

Supplementary Information
