# Supplementary material for: Conservation of oncofetal antigens on human embryonic stem cells enables discovery of monoclonal antibodies against cancer
Source: Sci Rep. 2018 Aug 2;8:11608. doi: 10.1038/s41598-018-30070-z (PMC6072701; doi:10.1038/s41598-018-30070-z)
Supplement: Supplementary file 2 — Supplementary Information [file 41598_2018_30070_MOESM2_ESM.pdf]

**Title**

# Conservation of oncofetal antigens on human embryonic stem cells enables discovery of monoclonal antibodies against cancer.

**Authors**

H.L. Tan [1,*], C. Yong [1], B.Z. Tan [1], W.J. Fong [1], J. Padmanabhan [1], A. Chin [1], V. Ding [1], A. Lau [1], L. Zheng [1], X. Bi [1], Y. Yuansheng [1], A. Choo [1,**]

[1.] Bioprocessing Technology Institute, Agency for Science, Technology and Research (A*STAR), Biopolis, Singapore
*Heng Liang Tan. Stem Cell Group, Bioprocessing Technology Institute, 20 Biopolis Way, #06-01, Centros, Singapore 138668. Email: tan_heng_liang@bti.a-star.edu.sg

## Supplementary Figure 1

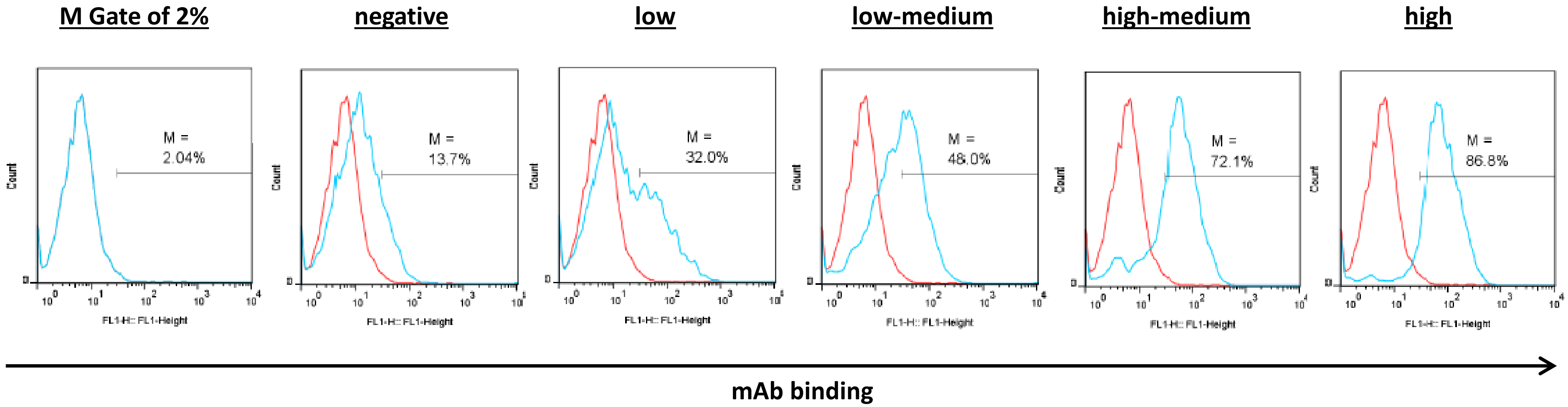


**Grading of A19's binding to cell lines.** The binding of the mAb is analysed by flow cytometry and binding is based on the population shift from the negative control with a M-gate of 2%. The grading of the binding is as follows: - negative binding represents < 20% cell population binding; -/+ low binding represents 20-40% cell population binding; + low-medium binding represents 40-60% cell population binding; ++ high-medium binding represents 60-80% cell population binding; +++ high binding represents > 80% cell population binding.

# Supplementary Figure 2

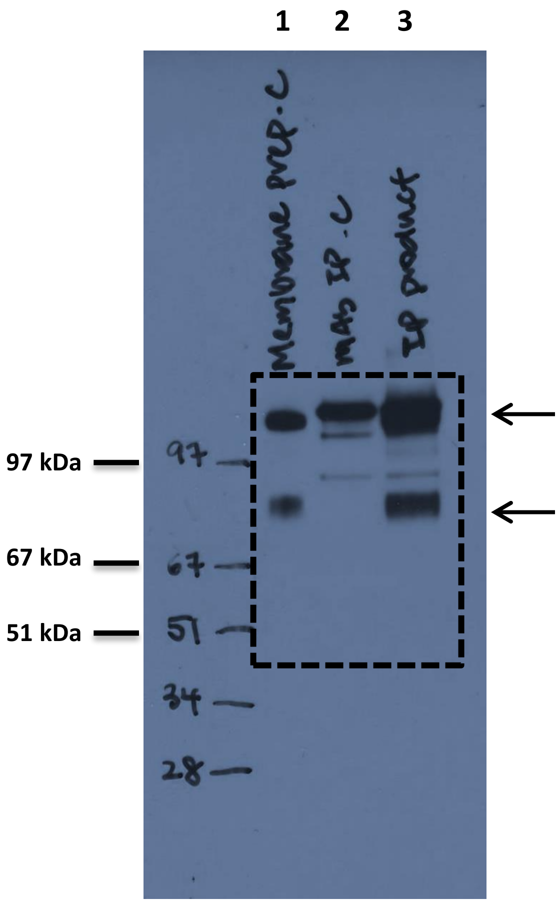


**Full length blot of Figure 1(A).** Western blot of protein samples immunoblotted (IB) with A19. Lane 1; membrane fraction of SKOV3. Lane 2; IP with mAb A19 only (negative control). Lane 3; IP product – the 2 antigen bands are indicated by the arrows and were excised from a parallel SDS Page gel and the antigen targets identified via MS. Figure was cropped to size as depicted by the dotted box for presentation purposes in Figure 1A.

# Supplementary Figure 3

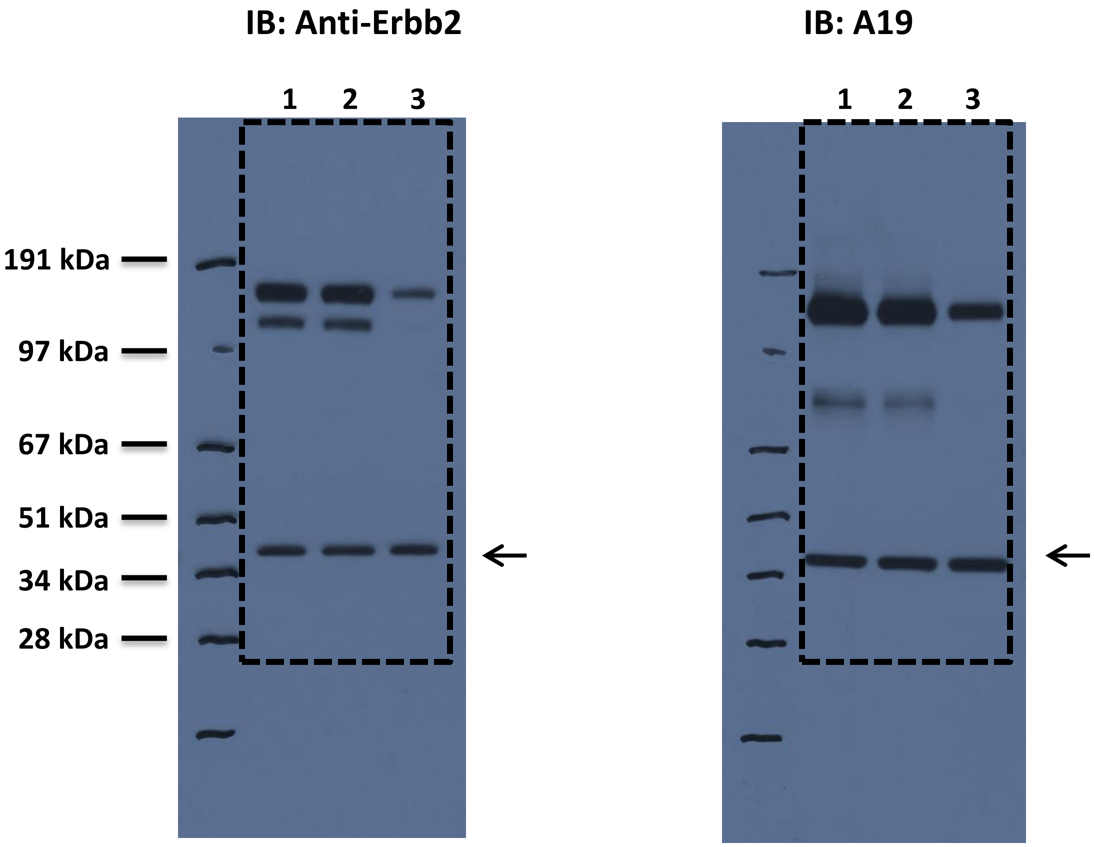


**Full length blots of Figure 1B.** Validation of the antigen target as Erbb2. Knock down of Erbb-2 was carried out using siRNA. Via western blot, both anti-Erbb-2 and A19 showed diminished binding, confirming that the antigen target is Erbb-2. Lane 1; lipofectamine control, Lane 2; scramble control, Lane 3; knockdown sample. The samples were normalised at 20 µg per well and actin was used as a loading control (black arrows). Figure was cropped to size as depicted by the dotted boxes for presentation purposes in Figure 1B.

# Supplementary Figure 4

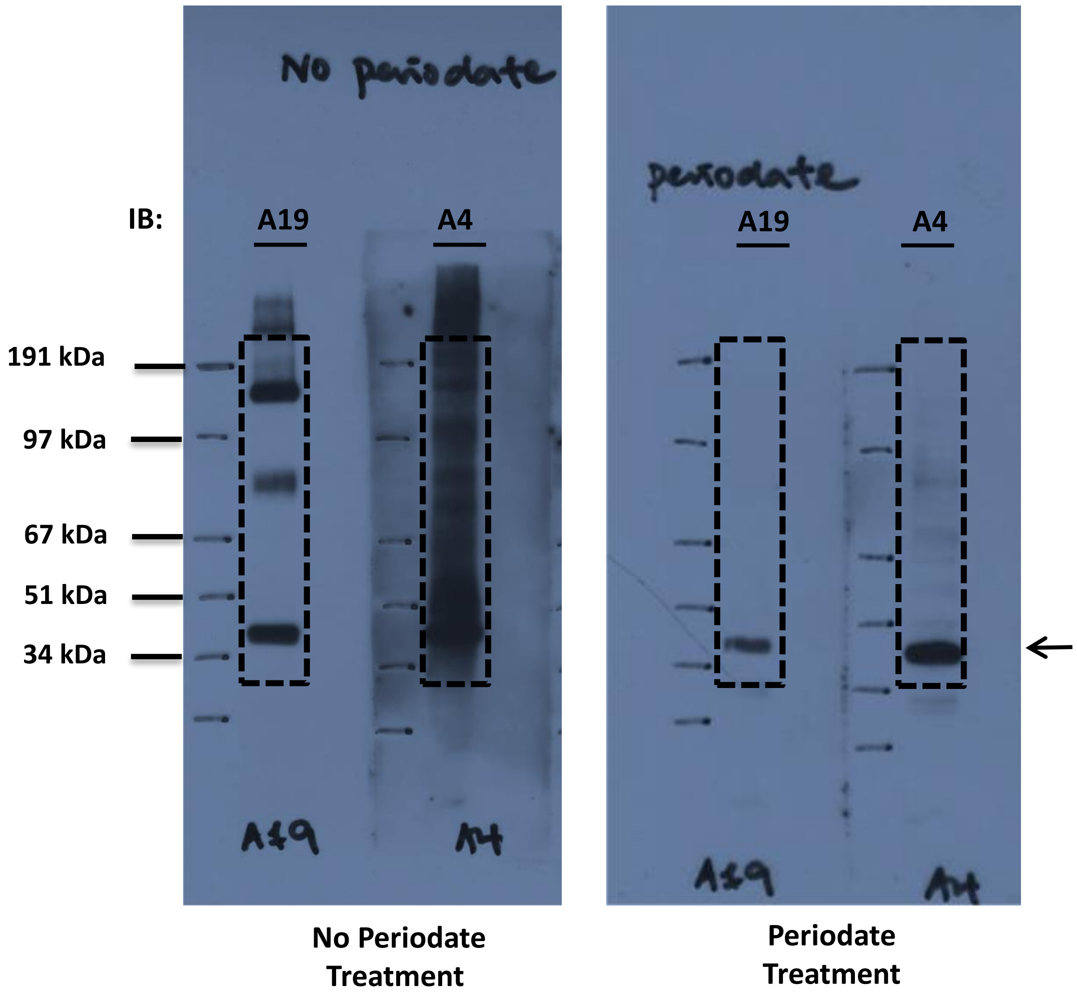


**Full length blots of Figure 2A.** SKOV3 membrane fraction was exposed to periodate oxidation and immuno-blotted with mAb A4 (positive control) and A19. Lanes were cropped as depicted by the dotted boxes and combined for presentation purposes in Figure 2A.

# Supplementary Figure 5

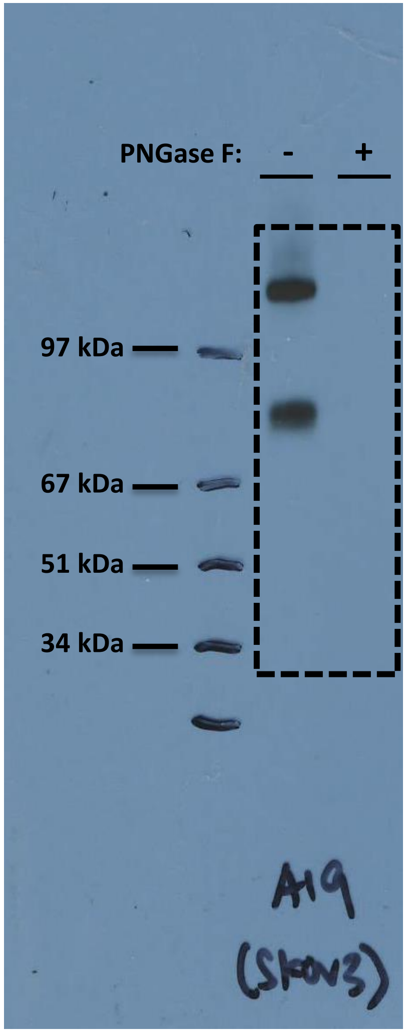


**Full length blot of Figure 2B.** SKOV3 membrane fraction was digested with PNGase F to remove N-glycans and western blot was carried out. Lanes were cropped as depicted by the dotted box for presentation purposes in Figure 2B.

## Supplementary Figure 6

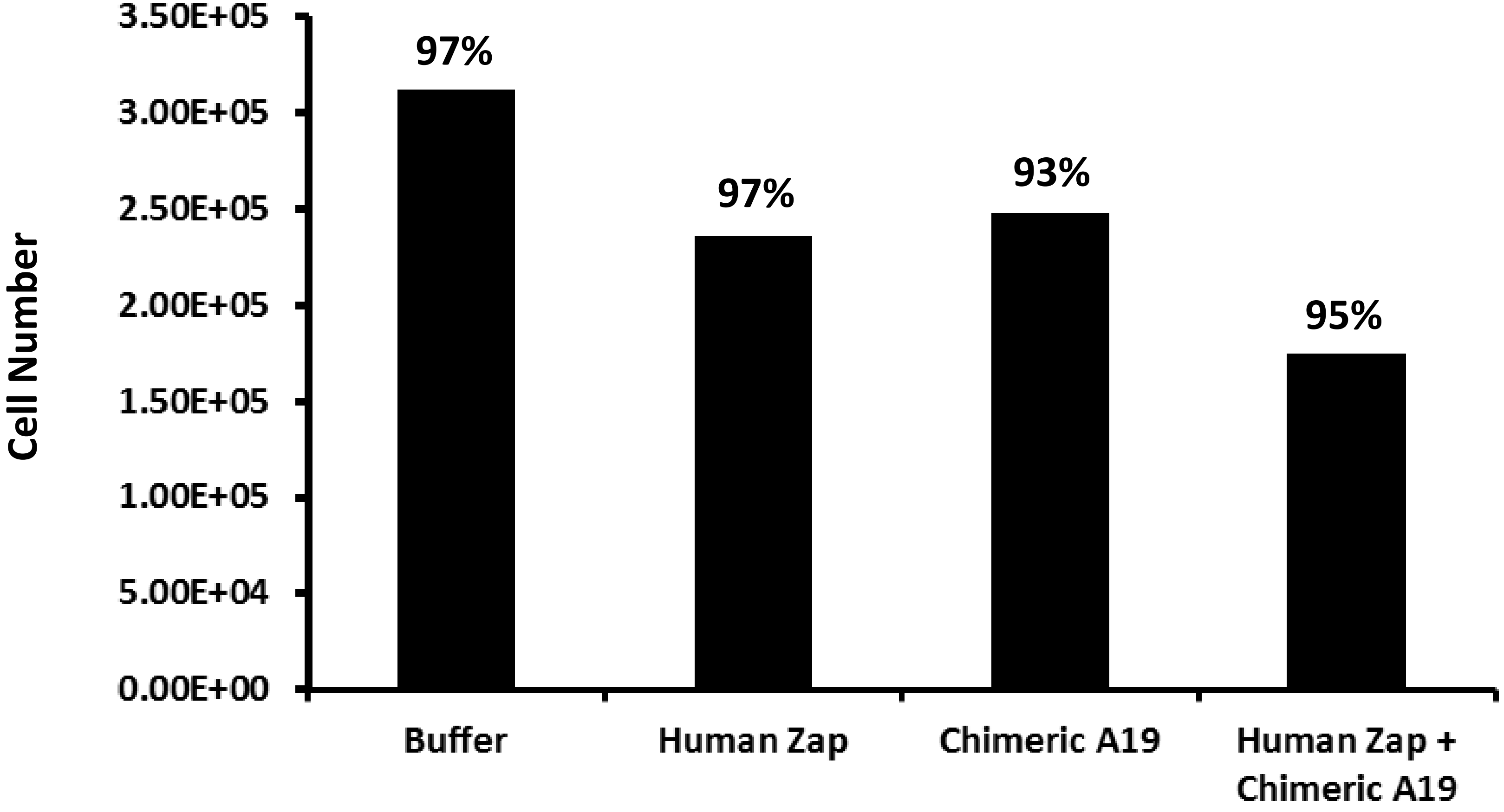


**Cell count and Viability Assay.** Cells in the various conditions were scaled-up accordingly to 24-wells each. 72 h post-treatment with the ADC complex, the number of cells and viability were determined. The viability of the cells in each condition remained high (as indicated above the bars).

## Supplementary Figure 7

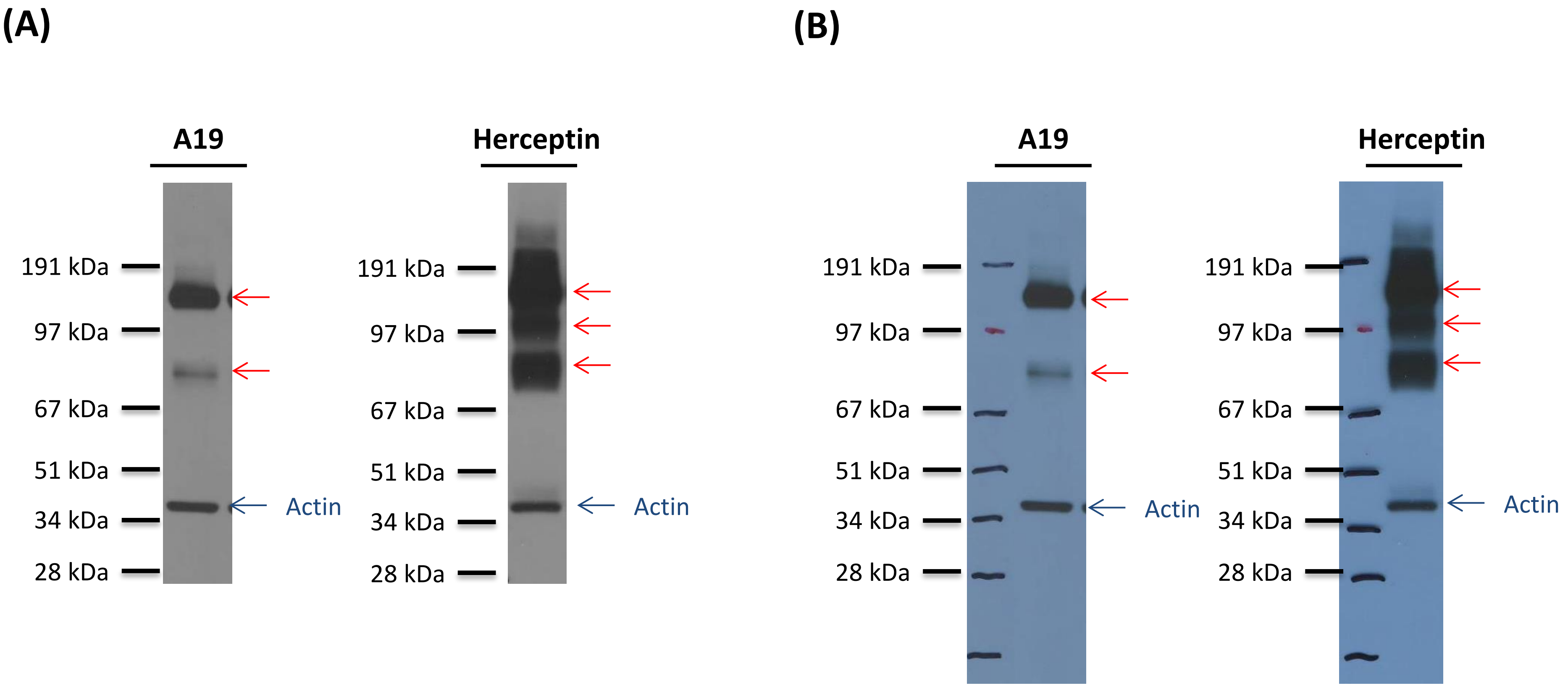


**Comparison of A19 with Herceptin.** (A) A19 binds to 2 isoform while Herceptin binds to 3 isoforms (as shown by the arrows). (B) Full length blots of Supplementary Figure 5A. Figures had been cropped to display protein bands > 28 kDa and molecular weight markers were digitally added. Figures were converted to grayscale using Microsoft PowerPoint.

**Supplementary Table 1**

**Binding of A19 and Herceptin to normal cells.** Live cells were incubated with the mAbs on ice for 45 mins, washed and stained with secondary antibody labelled with FITC. The binding of the mAbs is analysed by flow cytometry and based on the population shift from the negative control as follows: - negative binding; -/+ low binding; + low-medium binding; ++ high-medium binding; +++ high binding.

| **Cell type** | **Cell line** | **Binding** | |
|---|---|---|---|
| | | A19 | Herceptin |
| Normals | Renal | - | - |
| | Human Esophageal Epithelial Cells | -/+ | + |
| | Keratinocytes | - | + |
| | Adult Mesenchymal Stem Cells (aMSC) | - | - |
| | Red blood cells | - | - |
| | PBMCs | - | - |
| | Keratinocytes | - | + |
| | Pancreas duct | - | - |
